# Bronchodilatory effect of inhaled budesonide/formoterol and budesonide/salbutamol in acute asthma: a double-blind, randomized controlled trial

**DOI:** 10.1186/1471-2431-12-21

**Published:** 2012-03-07

**Authors:** Jenish J Arun, Rakesh Lodha, Sushil K Kabra

**Affiliations:** 1 Department of Pediatrics, All India Institute of Medical Sciences, New Delhi, Ansari Nagar 110029, India

**Keywords:** Acute asthma, Bronchodilatation, Formoterol, Salbutamol

## Abstract

**Background:**

There are no published studies that have compared bronchodilatory effect of inhaled budesonide/formoterol combination with budesonide/salbutamol delivered by metered dose inhaler with a spacer in acute exacerbation of asthma in children. We, therefore, compared the bronchodilatory effects of inhaled budesonide/formoterol (dose: 200 μg and 12 μg respectively) combination with budesonide (200 μg)/salbutamol (200 μg) administered by metered dose inhaler and spacer in children of 5-15 years with mild acute exacerbation of asthma [Modified Pulmonary Index Score (MPIS) between 6-8] in this double-blind, randomized controlled trial. The primary outcome was FEV1 (% predicted) in the two groups at 1, 5, 15, 30, 60 min after administration of the study drug.

**Results:**

We did not observe any significant differences in the % predicted FEV1 and MPIS between formoterol and salbutamol at various time points from 1 min to 60 min post drug administration. There was significant improvement in FEV1 (% predicted) from baseline in both the groups as early as 1 min after drug administration.

**Conclusions:**

Salbutamol or formoterol delivered along with inhaled corticosteroid by metered dose inhaler with spacer in children between 5-15 years of age with mild acute exacerbation of asthma had similar bronchodilatory effects.

**Trial Registration:**

ClinicalTrials.gov: NCT00900874

## Background

Bronchial asthma is one of the commonest chronic respiratory illnesses encountered in clinical practice in both adults and children. Asthma therapeutics can be broadly divided into long-term controllers and short-term reliever medications. Among inhaled bronchodilators, short acting beta agonists (SABA) like salbutamol and terbutaline are preferred in acute asthma exacerbations while inhaled corticosteroids (ICS) are the mainstay for long term treatment of asthma [1]. In moderate to severe persistent asthma, long acting beta agonists (LABA) like salmeterol and formoterol are added to ICS [1]. Efficacy and safety of LABA has been demonstrated in both adult and young asthmatics [2].

It is desirable to have a single inhaled medication that can be used as both reliever and controller; formoterol is likely to be one such agent as it has both rapid onset and long lasting action [3]. This will facilitate use of a single formulation (ICS + Formoterol) in children with persistent asthma. Few studies performed in adults with acute asthma exacerbation have demonstrated that formoterol was as efficacious and well tolerated as salbutamol [4-6]. Rodrigo et al. identified nine randomized controlled trials (576 patients; 3 trials included children) comparing formoterol with SABAs in acute exacerbation [4]; no significant difference was detected between formoterol and salbutamol in clinical and spirometric parameters.

In a systematic review conducted to assess the efficacy and safety of formoterol as reliever therapy in comparison to SABAs in adults and children with asthma, the authors reported that formoterol was similar to SABAs when used as a reliever, and showed a reduction in the number of exacerbations requiring a course of oral corticosteroids in adults [5]. Formoterol may have an advantage of longer duration of action as compared to SABAs. However, for children with asthma, there was insufficient information reported in the included trials to arrive at any conclusion on the safety or efficacy of formoterol as reliever [5].

There are few studies in children [6,7] which have compared formoterol and salbutamol delivered by different routes like nebulisation or dry powder inhalation in acute exacerbation. These studies concluded that both the drugs were equally efficacious in acute exacerbation. Bussamra et al. [8] compared terbutaline and formoterol delivered by dry powder inhaler performed in children with mild to moderate acute exacerbation of asthma; both treatments resulted in similar clinical and functional improvement without any adverse effects. LABA (including formoterol) montherapy is not indicated in patients with asthma and the LABAs should only be used in combination with ICS for moderate and severe persistent asthma patients who cannot be well controlled with ICS and SABA treatments.

There are no published studies that have compared budesonide/formoterol with budesonide/salbutamol delivered by metered dose inhaler with a spacer in acute exacerbation of asthma in children.

The objective of this study was to compare bronchodilatory effect of inhaled budesonide/formoterol (dose: 200 μg and 12 μg respectively) combination with budesonide (200 μg)/salbutamol (200 μg) administered by metered dose inhalers and spacer in children of 5-15 years with mild acute exacerbation of asthma; the evaluation were done only over a period of one hour after administration of medications.

## Methods

### Patients

Children of either sex between ages of 5-15 years with mild acute exacerbation of asthma were enrolled after obtaining written informed consent from the parents. Children with acute life threatening asthma, chronic respiratory illness and those who took salbutamol 6 h prior or those getting long acting β_2 _agonists were excluded.

### Study design

This double blind, randomized controlled trial was carried out in Pediatric out patient and Pediatric Chest Clinic (PCC) of a tertiary care hospital in north India. Randomization was done using computer-generated sequences by a scientist not involved in the conduct of this trial. Allocation was concealed with the use of sealed, opaque envelopes.

### Study drugs

We used Formoterol & budesonide combination MDI (6 μg of formoterol + 100 μg of budesonide per actuation) and placebo MDI in one group and salbutamol MDI (100 μg per actuation) and budesonide MDI (100 μg per actuation) in the other group. Lupin Limited (formoterol + budesonide combination MDI and placebo MDI) and Cipla Limited (salbutamol MDI and Budesonide MDI) supplied the MDIs. These pharmaceutical companies did not have any role in the trial except providing medications. For blinding, similar looking metered dose inhalers (MDIs) were labeled in pairs: A1, A2; B1, B2; C1, C2; D1, D2; E1, E2; F1, F2. As per random numbers patients were given drugs from these MDIs. The study staff as well as the subjects were blinded to the interventions as the canisters were similar in appearance and size; the original labels were removed from all of the canisters.

### Intervention

Children were administered 2 actuations of each MDI to receive either formoterol (12 μg) or salbutamol (200 μg) with a spacer; both groups received 200 μg budesonide. Participants were explained about the study, spirometry and inhalation of medicines with metered dose inhaler and spacer.

### Assessment

Mild acute exacerbation was defined as children presenting with increase in symptoms (cough, wheeze, breathlessness) for less than 7 days duration, no chest indrawing, and no difficulty in speech with Modified Pulmonary Index Score (MPIS) between 6-8 [9]. MPIS was calculated by the physician after observing the child for the following aparmeters: SpO2 at room air, use of accessory muscles, inhalational: exhalation ratio; wheezing; Heart rate and respiratory rate [9]. This evaluation could be completed within a minute. A baseline spirometry was done using portable spirometer (Superspiro MK2 Micro Medical Ltd, UK). FVC, PEFR, FEV1, FEF_50 _were recorded. Spirometry was repeated at 1, 5, 15, 30, and 60 min after administration of study drug. The patient was asked to perform spirometry thrice each time and the best of the effort was recorded. Similarly MPIS was calculated at these time points. In addition, data regarding any adverse effects like tremors, nausea, vomiting were recorded. Details of history and physical examination including symptoms with their duration, type of asthma, family history of allergy and medications were recorded in a structured performa; this was done after the initial evaluation was done and the child administered the study medications. If the child did not improve or showed worsening during study period, dose of salbutamol was administered using a MDI.

The study protocol was approved was by the AIIMS Institute Ethics Committee (Ref. No.: C/A-72/2.08.2008). The research was carried out in compliance with the Helsinki Declaration. The trial was registered at clinicaltrials.gov (*ClinicalTrials.gov Identifier: NCT00900874*).

### Outcome measures

The primary outcome variable was FEV_1 _(% predicted) in the two groups at 1, 5, 15, 30, 60 min after administration of the study drug.

Secondary outcome variables that were evaluated included the difference in Modified Pulmonary Index Score between the two groups, adverse effects like tremors, vomiting, palpitation, etc., number of patients requiring hospitalization at end of study period, and number of children who deteriorated during 1-h study period.

### Sample size

Sample size was calculated for equivalence. If there is truly no difference between control (Salbutamol) and experimental (Formoterol) groups, then 72 patients are required to be 80% sure that the 95% confidence interval will exclude a difference in mean FEV_1 _(% predicted) of more than 10 [(assuming SD of 15, based on our unpublished data and data from another study [8,10].

### Statistical methods

Data were managed in Microsoft Excel spreadsheet and analyzed by STATA software (StataCorp College Station, TX) before breaking the code. The baseline parameters between both the groups were compared. Mean and standard deviation were used to summarize the continuous variables. Inter-group and intra-group comparison of FEV_1_- percent predicted and Modified Pulmonary Index Score was done at 0, 1, 5, 15, 30 and 60 min. Chi-square test was used for comparing proportions and 't' test for continuous variables. A p value of < 0.05 was considered significant.

## Results

**Figure 1:**
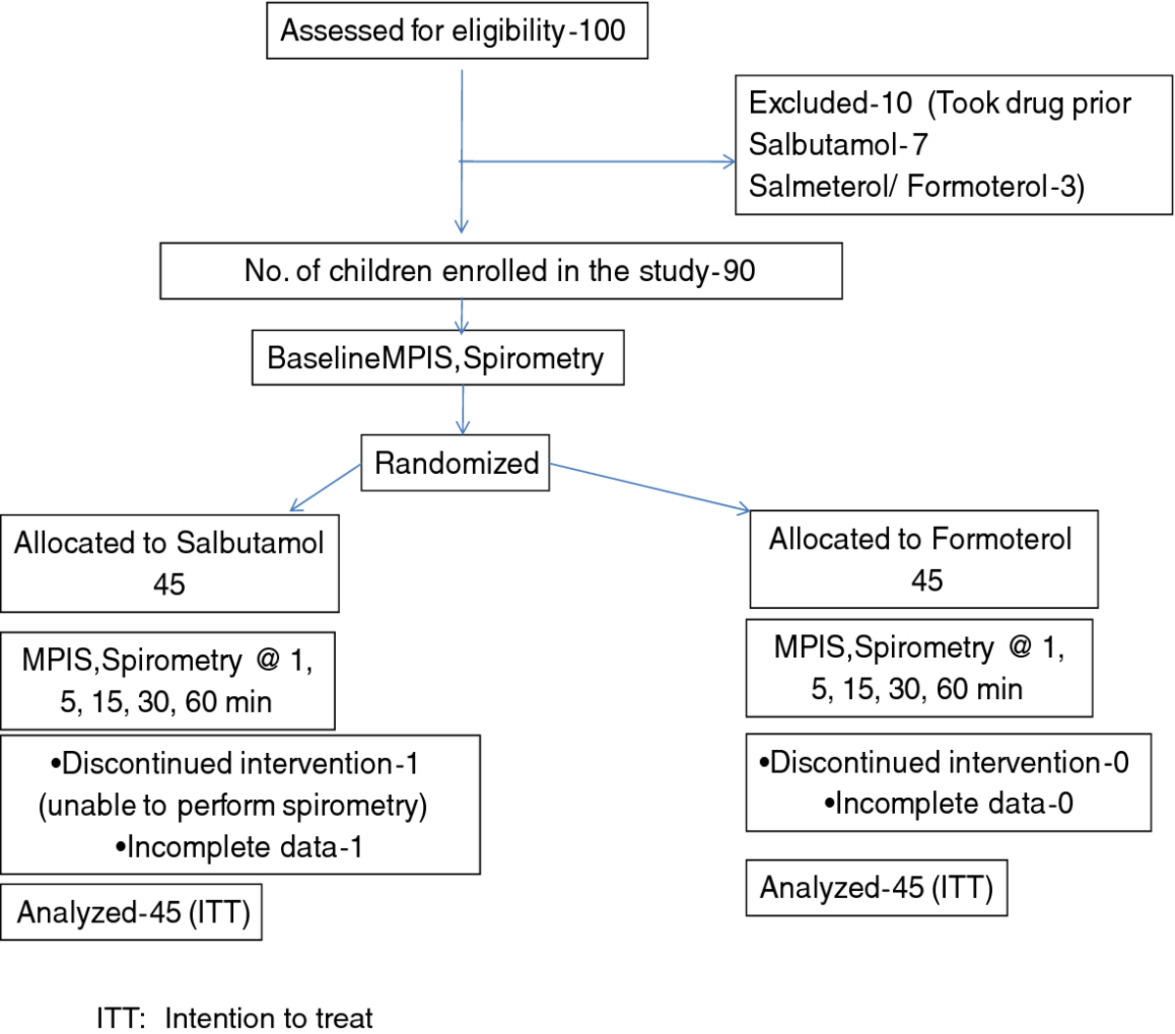
**Trial profile**. ITT: Intention to treat.

100 children of either sex, 5 to 15 years of age, with acute mild exacerbation of asthma attending Pediatric OPD or the Pediatric Chest Clinic were screened for enrolment in the study between May 2009 and April 2010. 10 of them were not enrolled as they had taken salbutamol or LABA prior to the screening. Ninety children were enrolled in the study and randomized to 2 groups- 45 to budesonide/salbutamol group (children received salbutamol and budesonide), and 45 to budesonide/formoterol group (children received formoterolbudesonide combination and placebo). Two patients in salbutamol group could not complete the study (one child could not perform spirometry and data were incomplete in the other patient). Trial profile is shown in Figure 1. No child failed to stabilize during the observation period.

The mean (SD) age of children in the budesonide/salbutamol group was 8.6 (2.3) years and 8.9 (2.1) years in the budesonide/formoterol group. The mean (SD) weight and height of children in budesonide/salbutamol group were 23.3 (6.3) kg and 127.2 (14.2) cm, respectively whereas in budesonide/formoterol group it was 25 (8.2) kg and 129 (13.5) cm, respectively. Presenting complaints like cough, breathlessness, night symptoms, type of asthma, frequency of exacerbation per year and family history of allergy or asthma were comparable between both the groups. The most common presenting symptoms were breathlessness, cough, night symptoms and wheeze in both the groups (Table 1).

**Table 1:** Baseline characteristics of the study subjects

	Budesonide/Salbutam ol group (n = 45)	Budesonide/Formoterol group (n = 45)
Age, years	8.6 ± 2.3	8.9 ± 2.13
Sex- male/female, n	33/12	30/15
Weight, kg	23.3 ± 6.3	25 ± 8.2
Height, cm	127.2 ± 14.2	129 ± 13.5
Duration of symptoms, days	6.0 ± 3.6	6.1 ± 4.1
Cough	44 (98%)	44 (98%)
Breathlessness	45 (100%)	41 (91%)
Night symptoms	27 (60%)	33 (73%)
Wheeze	24 (53%)	23 (51%)
Frequency of exacerbations	3 [2-6]	3 [2-6]
per year [median(IQR)]		
Type of asthma		
1. Mild intermittent	19 (42%)	18 (40%)
2. Mild persistent	15 (33%)	15 (33%)
3. Moderate persistent	10 (22%)	12 (27%)
4. Severe persistent	1 (2%)	0 (0%)
MPIS	7.3 ± 1.2	7.2 ± 1.0
FEV_1 _(% predicted)	58.2 ± 18.1; N = 43	55.9 ± 14.8
PEFR (% predicted)	55.5 ± 21.5; N = 43	58.6 ± 16.3
FVC (% predicted)	61.5 ± 28.3; N = 43	53.8 ± 20.8
FEF_50 _(% predicted)	65.9 ± 37.6; N = 43	70.6 ± 23.7
FEV_1_/FVC (% predicted)	84.9 ± 21.9; N = 43	91.4 ± 14.5

MPIS: Modified Pulmonary Index Score; Values are Mean ± SD, unless indicated

The type of asthma presenting with mild acute exacerbation in this study were predominantly intermittent or mild persistent type in both the groups (Table 1). Only one case of severe persistent asthma was enrolled in budesonide/salbutamol group. Presence of family history of allergy or asthma was present in 22 (49%) children in budesonide/salbutamol group and 21 (47%) in the budesonide/formoterol group.

The mean (SD) baseline MPIS score were 7.3 (1.2) and 7.2 (1.0) in the budesonide/salbutamol and budesonide/formoterol groups, respectively. The mean baseline spirometric parameters were similar in both the groups- FEV_1 _(% predicted) in the budesonide/salbutamol group at baseline was 58.2 (18.1)% while that of budesonide/formoterol group was 55.9 (14.8)%.

**Figure 2:**
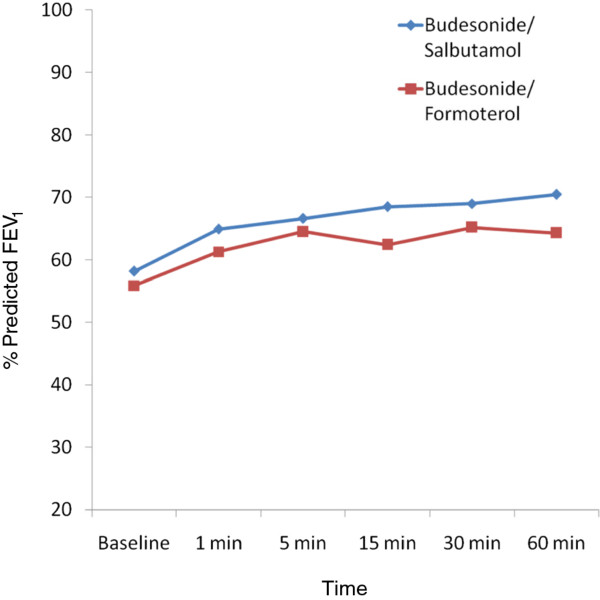
**% predicted FEV_1 _at different time points in the 2 groups**.

On comparing the post intervention values of percent predicted FEV1 between both the groups at 1, 5, 15, 30 and 60 min, there was no statistically significant difference at any of the time points and the difference between both the groups were always less than 10 (p > 0.05) (Table 2, Figure 2). The mean (SD) absolute change in FEV1 (% predicted) from baseline to 60 min was 12.2 (13.2) in the budesonide/salbutamol group while in the budesonide/Formoterol group, it was 8.3 (13.0) with a p value of 0.16.

**Table 2:** FEV_1 _(percent predicted) in the two groups at different time points

Time	Budesonide/Salbutamol group N = 43	Budesonide/Formoterol group N = 45	P value
Baseline	58.2 ± 18.1	55.9 ± 14.8	
1 min	64.9 ± 18.9	61.3 ± 14.7	0.32
5 min	66.6 ± 17.1	64.5 ± 18.2	0.58
15 min	68.5 ± 17.9	62.4 ± 16.1	0.09
30 min	69.0 ± 18.2	65.2 ± 17.9	0.33
60 min	70.5 ± 20.7	64.3 ± 16.4	0.12

Values are mean ± SD

Intragroup analysis showed significant improvement in FEV_1 _(% predicted) (p < 0.0001) at 1, 5, 15, 30, 60 min compared to baseline in both the groups. The percentage change in FEV_1 _from baseline at different time points were also similar (Table 3). Peak expiratory flow rates (PEFR), percentage predicted PEFR (Table 4) and forced expiratory flow-50 (FEF 50) and percentage predicted FEF_50 _of both the groups were similar at different time points during the study. Table 5 shows the percent change in PEFR from baseline at different time points. There was no significant difference between salbutamol and formoterol groups in other spirometric parameters also.

**Table 3:** Percentage change in FEV1 from baseline at different time points

Time	Budesonide/Salbutamol group N = 43	Budesonide/Formoterol group N = 45	P value
1 min	11.4 ± 26.8	12.9 ± 25.0	0.77
5 min	16.8 ± 28.5	19.4 ± 33.4	0.70
15 min	20.0 ± 31.6	16.7 ± 30.3	0.60
30 min	22.1 ± 35.9	20.8 ± 35.1	0.86
60 min	23.7 ± 34.3	19.3 ± 31.1	0.52

Values are mean ± SD

**Table 4:** PEFR (percent predicted) in the two groups at different time points

Time	Budesonide/Salbutamol group N = 43	Budesonide/Formoterol group N = 45	P value
Baseline	58.5 ± 21.5	58.6 ± 16.3	
1 min	63.4 ± 25.8	59.8 ± 16.9	0.43
5 min	67.4 ± 25.6	63.9 ± 14.2	0.42
15 min	67.2 ± 24.7	64.9 ± 16.7	0.60
30 min	68.6 ± 21.8	65.6 ± 14.8	0.46
60 min	70.2 ± 22.5	64.8 ± 15.8	0.19

Values are mean ± SD

**Table 5:** Percentage change in PEFR from baseline at different time points

Time	Budesonide/Salbutamol group N = 43	Budesonide/Formoterol group N = 45	P value
1 min	14.4 ± 30.9	7.8 ± 29.8	0.31
5 min	25.9 ± 35.7	18.2 ± 36.8	0.31
15 min	26.7 ± 43.7	21.0 ± 42.2	0.54
30 min	35.4 ± 54.9	21.3 ± 37.8	0.16
60 min	36.3 ± 50.8	19.7 ± 37.2	0.08

Values are mean ± SD

The Modified Pulmonary Index Scores of both the groups were similar at 1, 5, 15, 30 and 60 min of the study (Table 6).

**Table 6:** Modified Pulmonary Index Score (MPIS) in the two groups at different time points

Time	Salbutamol group N = 43	Formoterol group N = 45	P value
Baseline	7.3 ± 1.2	7.2 ± 1.0	
1 min	5.4 ± 1.6	5.4 ± 1.9	0.66
5 min	4.3 ± 1.5	4.6 ± 1.7	0.38
15 min	3.9 ± 1.6	4.1 ± 1.8	0.66
30 min	3.6 ± 1.6	3.5 ± 1.7	0.8
60 min	3.5 ± 1.6	3.6 ± 1.9	0.76

Values are mean ± SD

Only 3 of the total 90 children experienced tremors. Two of them belonged to salbutamol group while one was from formoterol group; this difference was not statistically significant. None of the children had vomiting or palpitations. No child deteriorated during the study period or required hospitalization.

## Discussion

In this double blind, randomized controlled trial, we compared the bronchodilatory action of budesonide/formoterol and budesonide/salbutamol in mild acute exacerbation of asthma. We observed that budesonide/formoterol had rapid onset of bronchodilatory action similar to that of budesonide/salbutamol in mild acute exacerbation of asthma in children 5-15 years of age. Both clinical and spirometric parameters at different time points after the administration of the drugs from 1 to 60 min showed no statistically significant difference in both the groups. Children within both the groups showed significant improvement in FEV1 as well as MPIS. There was no significant difference in side effects.

Few studies in children which compared formoterol with salbutamol or terbutaline delivered by different routes like nebulisation or dry powder inhalation in acute exacerbation also concluded that both the drugs are equally efficacious in acute exacerbation [6-8].

There are no published studies that have compared budesonide/formoterol with budesonide/salbutamol delivered by metered dose inhaler with spacer in acute exacerbation of asthma in children. Studies in adults comparing rapid bronchodilator response of formoterol and salbutamol suggested comparable results [11-13].

A recent study by Bussamra et al. compared formoterol (12 μg) delivered by aerolizer and terbutaline (0.5 mg) delivered by dry powder inhaler (Turbohaler) in mild to moderate acute exacerbation in children and reported similar rapid bronchodilator action [8]. In this study, all children received up to three doses of medications at interval of 20 min till they achieved predefined spirometric parameters. All participants received oral steroids. They concluded that both drugs had similar clinical and spirometric improvement, variations in FEV1 of 19.5% and 15.3% were observed in the formoterol and terbutaline groups, respectively. Rodriguez et al. compared formoterol and salbutamol in 50 children aged 5 to 12 years with acute asthma exacerbations of any severity. Children received either single dose of 25 μg formoterol fumarate by nebuliser or 3 doses of Salbutamol (Albuterol) every twenty minutes for one hour by nebuliser. Symptoms score, oxygen saturation and lung function testing recorded before and one hour after commencing treatment showed significant improvement [7]. The results of our study are in concordance with these studies even though we enrolled children with mild exacerbations and used single dose of either medication delivered by MDI and spacer.

The results of our study and other studies suggest that formoterol has rapid bronchodilator action similar to salbutamol or terbutaline and possibly can be used as rescue drug for acute exacerbations in children. We had earlier compared bronchodilator action of two long acting beta agonists (formoterol and salmeterol) at 60 min in a randomized controlled trial and observed similar improvement in FEV1 [14]. In this study, no comparisons were done in first 10 min of administration of the drugs. International guidelines recommend against monotherapy with LABA in the management of asthma [15].

However, there are few concerns regarding long-term, frequent use of LABAs. The extent of tolerance to β-adrenoceptor agonists is dependent on the dose and duration of treatment. A randomized, double-blind, placebo-controlled, crossover trial in adults by Haney and Hancox [16] assessed tolerance to the bronchodilator action of salbutamol during ongoing treatment with long-acting beta-agonist. They concluded that the bronchodilator response to salbutamol was significantly reduced in patients taking formoterol. Clinically relevant tolerance to rescue beta-agonist treatment may occur in patients treated with long-acting beta-agonists. Therefore, it is important to document that repeated doses of formoterol as rescue drugs will not affect response to treatment in acute exacerbation of asthma.

A systematic review evaluated the effects of the combination of LABA and inhaled corticosteroids versus a higher dose of inhaled corticosteroids on the risk of asthma exacerbations [17]. The authors concluded that combination of LABA and ICS was more effective in reducing the risk of exacerbations requiring oral corticosteroids than a higher dose of ICS in adolescents and adults; in children there was no significant reduction, but rather a trend towards an increased risk of oral steroid-treated exacerbations and hospital admissions was observed [17]. Hence, the issue of safety in children on long term use is yet to be resolved.

The strength of our study was that it was a double blind, randomized controlled trial with adequate sample size. We enrolled children with mild acute exacerbation of asthma. We used metered dose inhalers to deliver single dose (two actuations of MDI for each drug) as these devices are commonly used in the management of asthma. Unlike previous studies, we used a combination of ICS and formoterol. Clinical and spirometric parameters were monitored as early as 1-min post administration of the drug and frequent observations at 5, 15, 30 and 60 min were done.

There are few limitations of our study. As the study was done in children with only mild acute exacerbation, the results cannot be extrapolated to children with moderate to severe exacerbation. The evaluation were done only for an hour after administration; the advantages of formoterol + ICS versus salbutamol + ICS are more likely to be seen with longer follow-up periods, such as upto 3-12 h post-dosing.

## Conclusions

Results of our study suggest that budesonide/formoterol can be used as reliever using MDI and spacer in children with mild acute exacerbations of asthma. This approach may avoid carrying two different MDIs (salbutamol and ICS with formoterol), which may improve patient compliance. Formoterol should not be used as monotherapy for management of asthma. Before budesonide/formoterol is recommended routinely for management of acute exacerbations, issues like tolerance, long-term safety on repeated frequent use and cost effectiveness should be proven.

## Competing interests

The authors declare that they have no competing interests.

## Authors' contributions

JJA participated in study design, recruitment of patients and conduct of study; she also drafted the manuscript. RL was involved in the study design, conduct of study, analysis of data and drafting of manuscript. SKK contributed to the study dsign, its conduct and drafting of manuscript. All the authors read and approved the final manuscript.

## Pre-publication history

The pre-publication history for this paper can be accessed here:

http://www.biomedcentral.com/1471-2431/12/21/prepub
